# Potential factors, including activities of daily living, influencing home discharge for patients with putaminal haemorrhage

**DOI:** 10.1186/s12883-016-0539-x

**Published:** 2016-02-01

**Authors:** Shinichiro Maeshima, Sayaka Okamoto, Hideto Okazaki, Shiho Mizuno, Naoki Asano, Hirofumi Maeda, Mitsuko Masaki, Hiroshi Matsuo, Tetsuya Tsunoda, Shigeru Sonoda

**Affiliations:** Department of Rehabilitation Medicine II, School of Medicine, Fujita Health University, 424-1 Oodori-cho, Tsu, 514-1295 Japan; Nanakuri Memorial Hospital, Fujita Health University, 424-1 Oodori-cho, Tsu, 514-1295 Japan

**Keywords:** Activities of daily living, Discharge destination, Stroke, Functional independence measure

## Abstract

**Background:**

Convalescent rehabilitation wards assist stroke patients in acquiring skills for activities of daily living to increase the likelihood of home discharge. However, an improvement in activities of daily living does not necessarily imply that patients are discharged home. We investigated the characteristics of patients with putaminal haemorrhage who are discharged home following convalescence in rehabilitation wards.

**Methods:**

The sample comprised 89 patients (58 men and 31 women) with putaminal haemorrhage hospitalised in the convalescent rehabilitation ward of our hospital between August 2012 and July 2013. Their age ranged from 29 to 88 years (61.9 ± 11.9 years). The lesion occurred on the right side in 48 and on the left in 41 patients. The mean period from onset to hospitalisation in the convalescent rehabilitation ward was 30.8 ± 17.2 days, and the mean hospitalisation period was 70.7 ± 31.8 days. We examined age, sex, haematoma volume, duration from onset to hospitalisation, neurological symptoms, cognitive function, functional independence measure, number of cohabitating family members and whether the patient lived alone before stroke, and the relationship among these factors and discharge destination (home or facility/hospital) was assessed.

**Results:**

The discharge destination was home for 71 and a facility or hospital for 18 patients. Differences were observed in age, haematoma volume, neurological symptoms, cognitive function, functional independence measure score on admission and discharge, number of cohabitating family members and whether the patient lived alone before stroke for patients discharged home. Patients who required long-term care and were discharged home were more likely to be living with family members who were present during daytime. Home discharge was possible if functional independence measure score was ≥70 at the time of discharge for motor items and ≥24 for cognitive items, even if a patient lived alone before stroke.

**Conclusions:**

Although the presence of cohabitating family members was important, the factor most strongly influencing home discharge was the patient’s activities of daily living status at the time of discharge. For patients who lived alone before stroke, physical and cognitive functions must be maintained for them to be discharged home after rehabilitation.

## Background

Putaminal haemorrhage accounts for 30–40 % of all cerebral haemorrhages and is responsible for various neurological symptoms, including motor paralysis, [1]. Its prognosis varies according to factors such as age, neurological severity, site and size of haematoma, complications, and choice of treatment [2]. Therefore, rehabilitation plays a major role in the improvement of activities of daily living (ADL) in patients with putaminal haemorrhage (stroke) [3, 4].

In convalescent rehabilitation wards^1^, patients undergo intensive treatment in the early stages after stroke to help decrease ADL impairment, with the expectation that it will help speed up recovery [5]. However, despite reaching the same level of ADL, some stroke patients are able to be discharged home after rehabilitation, while others are not. Various factors influence discharge destination including family structure, number of family members, social background and pathology. Moreover, the recent increase in lifespan, geographic separation of nuclear families and number of single adults in Japan have led to annual increases in the number of elderly individuals living alone. This makes it difficult to allow patients to be discharged home following stroke [6]. Here we investigated the factors influencing discharge from convalescent rehabilitation wards according to the severity of ADL in patients with putaminal haemorrhage.

## Methods

Lesions of the putamen were observed in 126 of 328 patients with cerebral haemorrhage transferred to the convalescent rehabilitation ward of our hospital between August 2012 and July 2014. Of these, 89 patients (58 men, 31 women) with new-onset putaminal haemorrhage were included. Patients were excluded if they did not experience hypertensive cerebral haemorrhage, if they had recurrent or premorbid mental illness or neurological disease or if no image data was available from the acute phase. The age ranged from 29 to 88 years (61.9 ± 11.9 years). The mean period from onset to hospitalisation in the convalescent rehabilitation ward was 30.8 ± 17.2 days, and the mean period of hospitalisation was 70.7 ± 31.8 days. The lesion occurred on the right side in 48 and on the left in 41 patients.

Age, duration from onset to hospitalisation, duration from onset to the initiation of rehabilitation, lesion side, haematoma volume and clinical symptoms were assessed. Haematoma volume was measured as major axis × minor axis × height of the haematoma on computed tomography; its value was obtained by multiplying the total volume by half (mL) [7]. The Canadian Neurological Scale (CNS) and Mini-Mental State Examination (MMSE) were used to assess neurological severity [8] and cognitive function [9], respectively. The CNS includes the following components: comprehension, level of consciousness, speech and motor function (face, arm and leg). Scores submitted from each domain section are added to provide a total score out of a possible 11.5. Lower scores are representative of increasing severity. The MMSE, with a maximum total score of 30, consisted of five subtests including orientation, registration, calculation, recall and language. ADL was assessed on admission and at the time of discharge using the functional independence measure (FIM) [10], which is an 18-item ordinal measure of disability. Patients were assessed for each item on a 7 point ordinal scale that ranges from complete dependence (value = 7) to complete independence (value = 1). Total FIM scores range from 18 (lowest) to 126 (highest). Two sub-scales (motor and cognition) can be obtained by adding the 13 motor items (range, 13**–**91 points) and the five cognitive items (range, 5**–**35 points). Cognitive items of FIM scores were measured as an indicator of cognitive function.

At the time of discharge, factors such as whether the patient was discharged home, feeding status (regular diet or dysphagia diet/tube feeding), number of cohabitating family members and number of caregivers required during daytime were examined. Patients were divided into the following two groups based on discharge destination: a) those discharged home (home group) and b) those discharged to a facility or hospital (other group). Differences in the factors influencing this decision were subsequently investigated. This is an observational study for rehabilitation outcome of stroke patients. This research was conducted with the approval of Institutional Review Board committee of our hospital (Fujita Health University, No. 15-134), and the study was thoroughly explained to the patients before consent was received.

JMP version 8.02 was used for statistical processing. The Mann–Whitney U-test was used to test for unpaired differences between the two groups, and the chi-squared test was used as a test for independence of two factors. The area under the receiver operating characteristic (ROC) curve (AUC) was calculated.

## Results

The discharge destination was home for 71 and a facility or hospital for 18 patients. Differences were observed in patient discharge destination, haematoma volume, neurological symptoms and cognitive function on admission, duration of hospitalisation, FIM score on admission and at the time of discharge and feeding status (Table 1).

**Table 1:** Comparison between patients who could go home or not

	Home *n* = 71	Others *n* = 18	*p* value
Age, years old	61 (29–82)	68.5 (36–88)	0.0534
Gender, n, male/female	47/24	11/7	0.6876
Lesion side, n, Right/Left	38/33	10/8	0.8770
Haematoma volume (mL)*	17.5 (4.7–122.5)	40.1 (5–109.6)	0.0447
Duration from onset to admission (days)	26 (7–124)	36 (10–58)	0.1085
Canadian Neurological Scale (/11.5)***	6.5 (3–11.5)	3 (1.5–10.5)	0.0009
Mini-Mental State Examination (/30)*	25 (0–30)	18 (9–29)	0.0416
Length of Stay (days)**	74 (5–119)	89.5 (35–168)	0.0027
Functional independence measure score on admission (/126)***	64 (20–126)	30.5 (18–65)	<0.0001
Functional independence measure motor score on admission (/91)***	41 (13–91)	17.5 (13–40)	<0.0001
Functional independence measure cognitive score on admission (/35)***	24 (5–35)	12.5 (5–32)	0.0004
Functional independence measure score at discharge (/126)***	105 (45–126)	56.5 (18–105)	<0.0001
Functional independence measure motor score at discharge (/91)***	74 (29–91)	41 (13–72)	<0.0001
Functional independence measure cognitive score at discharge (/35)***	30 (8–35)	14.5 (5–33)	<0.0001
Nutrition intake, n, Regular food/Dysphagia diet***	62/9	8/10	0.0002
Number of family members living together	2 (0–5)	1 (0–4)	0.1692
Number of family members during daylight	1 (0–2)	0 (0–2)	0.3819
Family structure before stroke, n, living alone/living together	15/56	6/12	0.2899

*p < 0.05, **p < 0.01, ***p < 0.005, Median (range)

**Fig. 1:**
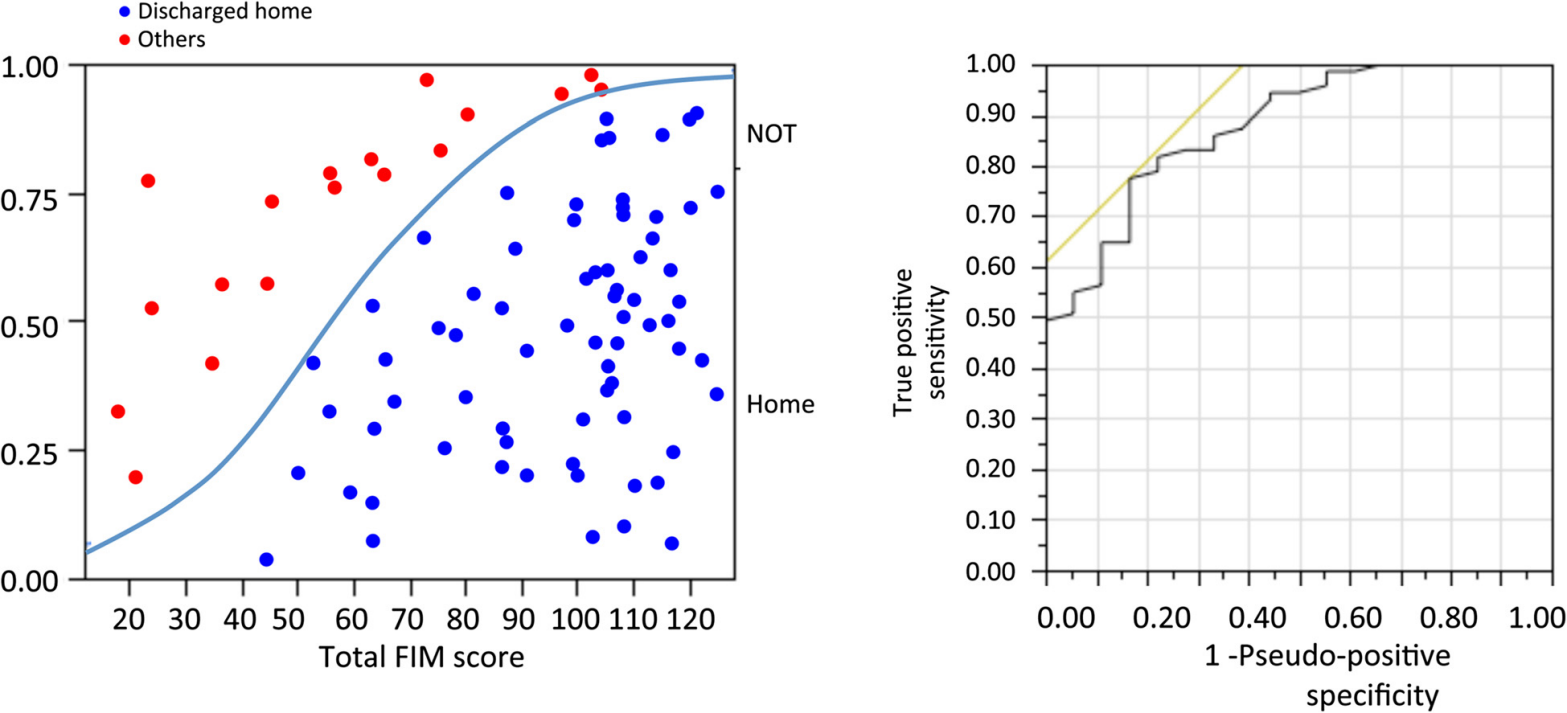
Correlation between discharge destination and functional independence measure score at discharge. The receiver operating characteristic curve shows functional independence measure score for predicting discharge destination. The area under the receiver operating characteristic curve for functional independence measure score was 0.877. Using a threshold of 82 for functional independence measure score, the sensitivity was 77.5 % and the specificity was 85.3 %. Total functional independence measure scores range from 18 (lowest) to 126 (highest)

Figure 1 shows the correlation between discharge destination and FIM score at discharge. The ROC curve shows FIM score for prediction of destination. The AUC for FIM score was 0.877. Using a threshold of 82 for FIM score, the sensitivity was 77.5 % and the specificity was 85.3 %.

**Fig. 2:**
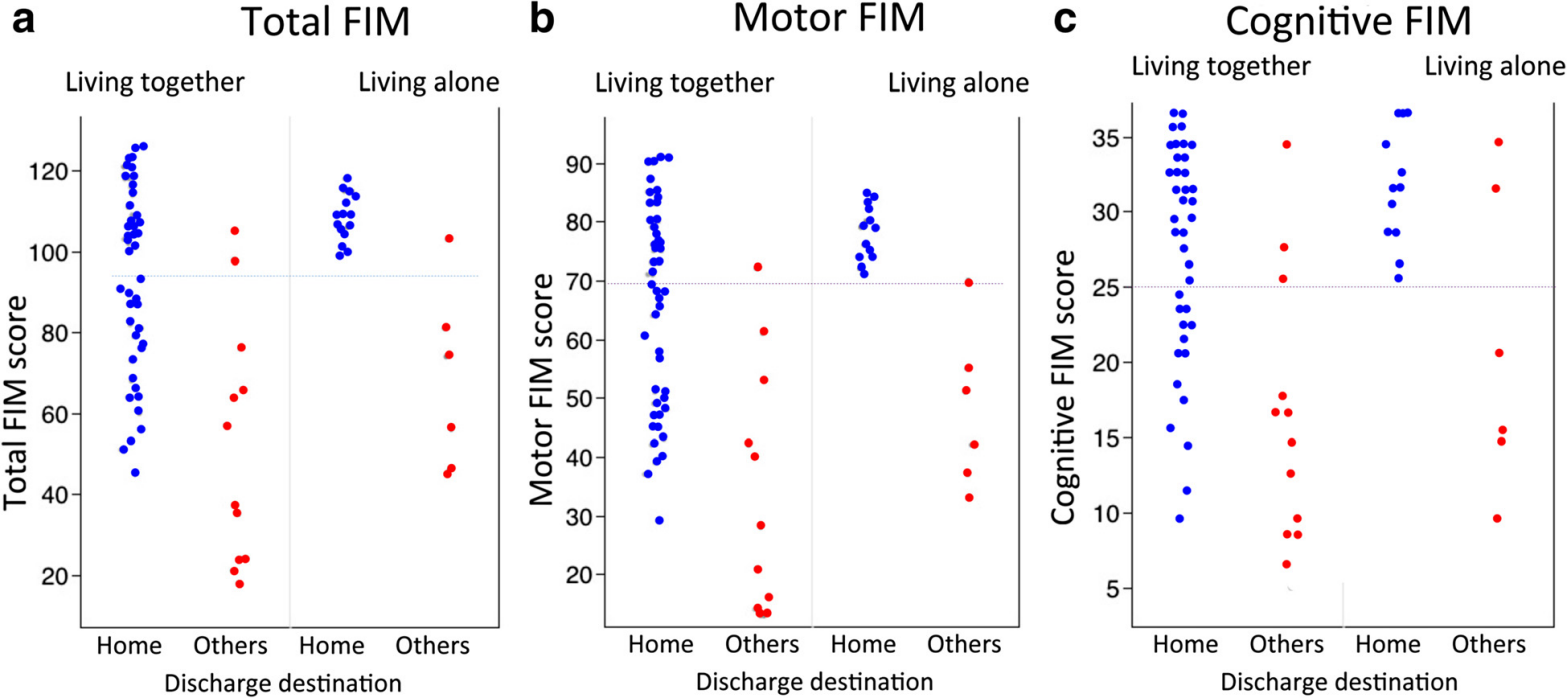
Patients who were discharged home according to whether or not they lived with family before stroke, and the score for each functional independence measure item. Total functional independence measure scores range from 18 (lowest) to 126 (highest) (**a**). Motor functional independence measure scores range from 13 (lowest) to 91 (highest) (**b**). Cognitive functional independence measure scores range from 5 (lowest) to 35 (highest) (**c**)

Fifteen of 21 patients (71.4 %) who had been living alone and 56 of 68 patients (82.4 %) who had been living with family before stroke were discharged home. Figure 2 shows patients who were discharged home according to whether or not they lived with family before stroke and the score for each FIM item. Even if a patient lived alone before stroke, home discharge was possible if FIM score was ≥70 at the time of discharge for motor items and ≥24 for cognitive items. However, living alone after discharge was difficult if either of these scores dropped below the aforementioned values (Fig. 2). As an exception, a 71-year-old man who lived alone before stroke was admitted to the geriatric health services facility, even though he was capable of living independently. Eighteen of 68 patients (82.4 %) who had been living with family before stroke were not discharged home. Most of them had low FIM scores. Two patients who were approximately 30 years old and had FIM scores of >100 were sent to a prefectural rehabilitation facility for vocational training.

## Discussion

In addition to patient factors such as age, sex, lesion side, cognitive function and reduced physical ability, the discharge destination of stroke patients is influenced by social factors such as the presence or absence of a caregiver or spouse and financial difficulties [11–15]. Few previous studies have focused on the home discharge on patients with cerebral haemorrhage [16]. In our study, we investigated factors such as haemorrhage type, haematoma volume, neurological symptoms and cognitive function in patients with putaminal haemorrhage. Our results showed that in comparison with the home group, the facility/hospital (i.e. other) group had a more severe type and volume of haematoma, neurological symptoms, poorer cognitive function and lower FIM score on admission and at the time of discharge. Moreover, many patients were unable to follow a regular diet and also tended to be hospitalised for longer durations. Meijer et al. [11] reported that the probability of home discharge was high in the presence of functional factors such as cohabitants, young age, mild paralysis, and independent ADL. In clinical settings, there may be instances where patients can be discharged home and some instances where they cannot.

We examined underlying factors that influenced the decision of home discharge according to the severity of ADL at the time of discharge. In the present study, the majority of patients with independent ADL were discharged home, barring special circumstances. In contrast, although patients who require much assistance were hospitalised, with the expectation of those who successfully completed convalescent rehabilitation, they were ultimately discharged to facilities or hospitals without obtaining the desired level of ADL. Nishio et al. [12] found that severe cognitive impairment and comorbidities were characteristic of patients who were not discharged home. They concluded that it was extremely difficult to provide intensive rehabilitation to these patients. However, the patient’s family members wanted his/her ADL to exceed that before stroke, despite little improvement in ADL following admission. These patients required daytime nursing care and supervision. The presence of caregivers and family greatly influenced the decision to select home discharge due to difficulties associated with those who lived alone before stroke. Caregiving is stressful, and caregivers often experience a variety of individual, interpersonal and organizational issues in managing stroke-related deficiencies [13]. Guidance provided directly to family members during hospitalisation can encourage them to understand the patient’s pathology and caregiving skills required [14, 15]. At present, rehabilitation successfully delivers elements of self-management support to stroke survivors and their caregivers, yielding improved outcomes [17]. Moreover, when a patient is transferred to a convalescent rehabilitation ward, it is essential that doctors re-establish the appropriateness of home discharge by assessing the family’s willingness to be actively involved in the patient’s care, including comparing the patient’s ADL at the time of discharge with their predicted ADL, assessing the family’s development of caregiving skills and ensuring that the family completes paperwork for long-term care insurance services.

In a study focusing on new-onset stroke, Massicci et al. [18] reported that living alone before a stroke negatively influenced home discharge. In the present study, we also found that discharge destination differed on the basis of whether patients lived alone before stroke, regardless of the level of ADL. A level of independent mobility at the time of discharge and absence of severe cognitive impairment reportedly are characteristics of patients who are discharged home [19, 20]. The limitation of this study was as follows: although we evaluated cognitive function using brief rating scales, an extensive neuropsychological examination would be required. The findings of this study suggest that it is important to maintain physical and cognitive functions in patients who lived alone before stroke and were discharged home after rehabilitation.

## Conclusions

We investigated the characteristics of patients with putaminal haemorrhage who were discharged home following convalescence in rehabilitation wards. Although the presence of cohabitating family members was important, the factor most strongly influencing home discharge was the patient’s ADL status during discharge. For patients who lived alone before stroke, physical and cognitive functions must be maintained for them to be discharged home after rehabilitation.

## Abbreviations

ADL: activities of daily living
AUC: area under the receiver operating characteristic curve
CNS: Canadian Neurological Scale
FIM: functional independence measure
MMSE: Mini-Mental State Examination
ROC: receiver operating characteristic

## Footnotes

1 Convalescent rehabilitation wards: In Japan, rehabilitation is conducted at the convalescent stage based on the medical insurance system. Inpatient treatment is carried out for a maximum of 6 months in rehabilitation hospitals for strokes, starting within 2 months of onset. During this time, remuneration for medical treatment can be calculated for up to a total of nine 20-min units (3 h) per day of physical, occupational and speech therapy.

## References

[1] Iwabuchi T (1990). Clinical study of putaminal hemorrhage in Tohoku Districts in Japan-A cooperative study. Jpn J Stroke..

[2] Last J, Perrech M, Denizci C, Dorn F, Kessler J, Seibl-Leven M (2015). Long-term functional recovery and quality of life after surgical treatment of putaminal hemorrhages. J Stroke Cerebrovasc Dis..

[3] Lehmann JF, Delateur BJ, Fowler RS, Wartren CG, Arnhold R, Schertzer G (1975). Stroke rehabilitation: outcome and prediction. Arch Phys Med Rehabil..

[4] Niki R (1982). Early prediction of the outcome of stroke rehabilitation. Jpn J Rehabil Med..

[5] Miyai I, Sonoda S, Nagai S, Takayama Y, Inoue Y, Kakehi A (2011). Results of new policies for inpatient rehabilitation coverge in Japan. Neurorehabil Neural Repair..

[6] Watabe N, Fujii Y (2013). Evaluation of stroke patients living alone. Jpn J Stroke..

[7] Kothari RU, Brott T, Broderick JP, Barsan WG, Sauerbeck LR, Zuccarello M (1996). The ABCs of measuring intracerebral hemorrhage volumes. Stroke..

[8] Côté R, Hachinski VC, Shurvell BL, Norris JW, Wolfson C (1986). The Canadian Neurological Scale: a preliminary study in acute stroke. Stroke..

[9] Folstein MF, Folstein SE, McHugh PR (1975). Mini-Mental State: a practical method for grading the cognitive state of patients for clinician. J Psychiatr Res..

[10] Granger CV, Hamilton BB, Linacre JM, Heinemann AW, Wright BD (1993). Performance profiles of the functional independence measure. Am J Phys Med Rehabil..

[11] Meijer R, van Limbeek J, de Haan R (2006). Development of the stroke-unit discharge guideline: choice of assessment instruments for prediction in the subacute phase post-stroke. Int J Rehabil Res..

[12] Nishio D, Hirano Y, Ito S, Kurata M, Kigawa H, Osawa A (2010). A study on the outcome and clinical features of stroke patients with severe disability in a convalescent rehabilitation ward. Jpn J Stroke..

[13] Grant JS, Hunt CW, Steadman L (2014). Common caregiver issues and nursing interventions after a stroke. Stroke..

[14] Maeshima S, Ueyoshi A, Osawa A, Ishida K, Kunimoto K, Shimamoto Y (2003). Mobility and muscle strength contralateral to hemiplegia from stroke: Benefit from self-training with family support. Am J Phys Med Rehabil..

[15] Hirano Y, Maeshima S, Osawa A, Nishio D, Takeda K, Baba M (2012). The effect of voluntary training with family participation on early home discharge in patients with severe stroke at a convalescent rehabilitation ward. Eur Neurol..

[16] James ML, Grau-Sepulveda MV, Olson DM, Smith EE, Hernandez AF, Peterson ED (2014). Insurance status and outcome after intracerebral hemorrhage: findings from Get with the guidelines-stroke. J Stroke Cerebrovasc Dis..

[17] Parke HL, Epiphaniou E, Pearce G, Taylor SJ, Sheikh A, Griffiths CJ (2015). Self-management support interventions for stroke survivors: a systematic meta-review. PloS One..

[18] Massucci M, Perdon L, Agosti M, Celani MG, Righetti E, Recupero E (2006). Italian Cooperative Research (ICR2): prognostic factors of activity limitation and discharge destination after stroke rehabilitation. Am J Phys Med Rehabil..

[19] Terai S, Miyamoto H, Nabeshima A (2008). A comparative study of patient discharge disposition in a convalescent rehabilitation unit: an analysis of cerebrovascular disorder and disuse syndrome cases. Jap J Rehabil Med..

[20] Kanayama T, Ohira Y, Nishida M, Nagaki T, Sakamoto M, Madoba K (2008). Characters of home discharge patients in a convalescence rehabilitation ward. Rigakuryoho Kagaku..

